# Supplementary material for: Mendelian Randomization Reveals Potential Causal Relationships Between DNA Damage Repair-Related Genes and Inflammatory Bowel Disease
Source: Biomedicines. 2025 Jan 19;13(1):231. doi: 10.3390/biomedicines13010231 (PMC11761251; doi:10.3390/biomedicines13010231)
Supplement: Supplementary file 1 [file biomedicines-13-00231-s001.zip › Supplementary figures.pdf]

Supplementary material

Supplementary figures

Figure S1. Leave-one-out sensitivity analyses of the SNPs represented the encode protein of DDR-related genes and Inflammatory bowel disease.

Figure S2. Leave-one-out sensitivity analyses of the SNPs represented the encode protein of DDR-related genes and ulcerative colitis.

Figure S3. Associations of genetically predicted DDR-related genes methylation with inflammatory bowel disease in Mendelianrandomization analysis.

Figure S4. Associations of genetically predicted DDR-related genes methylation with Crohn's disease in Mendelianrandomization analysis.

Figure S5. Associations of genetically predicted DDR-related genes methylation with ulcerative colitis in Mendelianrandomization analysis.

Figure S1. Leave-one-out sensitivity analyses of the SNPs represented the encode protein of DDR-related genes and Inflammatory bowel disease.

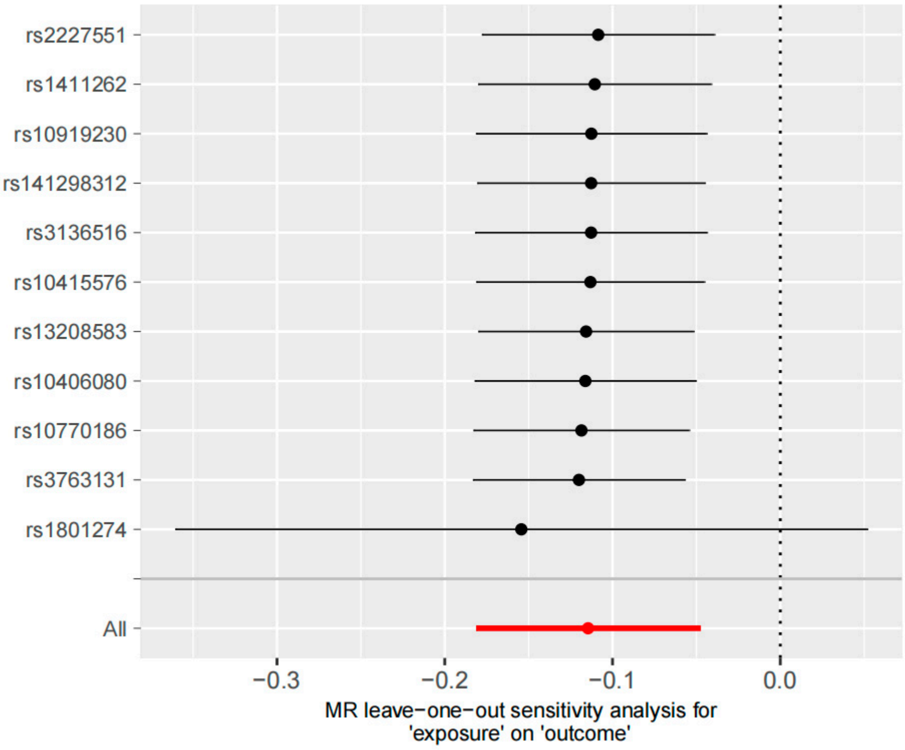

The estimated causal effect is shown for each excluded SNP and the overall estimate using all the SNPs is shown in red. The error bars represent the 95% confidence intervals.

Figure S2. Leave-one-out sensitivity analyses of the SNPs represented the encode protein of DDR-related genes and ulcerative colitis.

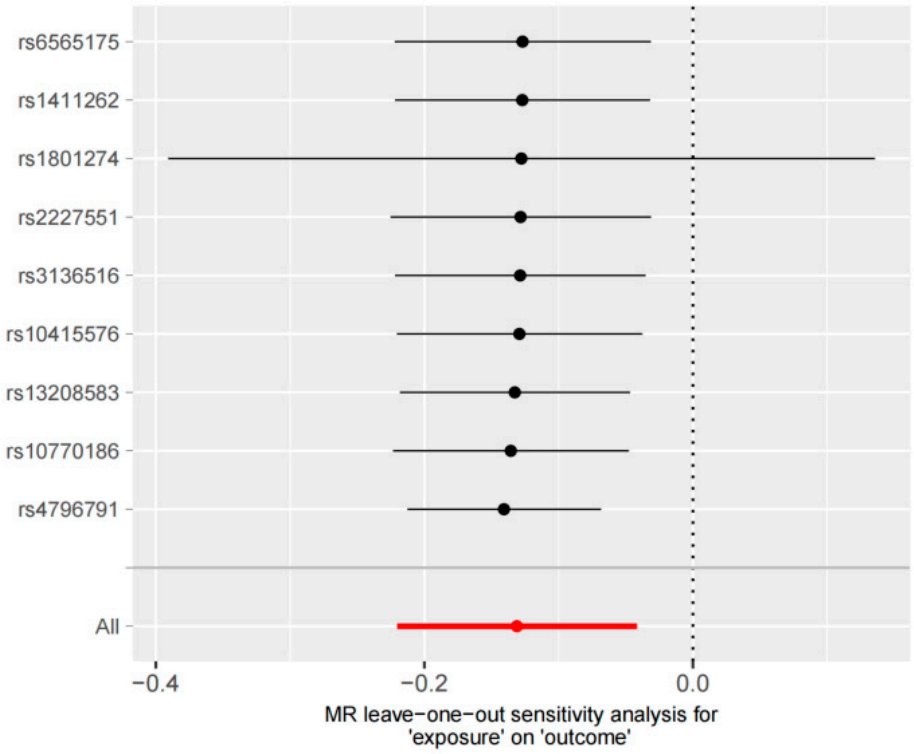

The estimated causal effect is shown for each excluded SNP and the overall estimate using all the SNPs is shown in red. The error bars represent the 95% confidence intervals.

Figure S3. Associations of genetically predicted DDR-related genes methylation with inflammatory bowel disease in Mendelian randomization analysis.

Disease	Gene	Methylation probe	Instrumental variant	mQTL size effect	β coefficient	OR(95%CI)	P_{SMR} value	Colocalization
IBD								
	ACTB	cg05971148	rs852389	−0.208	−0.399	0.67(0.55−0.82)	7.12e−05	0.000
	APAF1	cg10957001	rs2288713	−1.959	−0.035	0.96(0.94−0.99)	3.04e−03	0.518
	ASCC2	cg02474858	rs737911	−0.293	0.221	1.25(1.09−1.42)	9.31e−04	0.919
	BID	cg07473201	rs8190289	−1.272	0.096	1.10(1.03−1.17)	2.32e−03	0.694
		cg13346441	rs8190289	−1.608	0.076	1.08(1.03−1.13)	2.04e−03	0.694
		cg09896131	rs8190289	−1.132	0.108	1.11(1.04−1.19)	2.51e−03	0.694
	BRD2	cg26264318	rs580962	0.267	0.271	1.31(1.15−1.50)	5.37e−05	0.001
		cg26953232	rs580962	0.230	0.314	1.37(1.17−1.61)	1.11e−04	0.001
		cg17451586	rs580962	0.249	0.291	1.34(1.16−1.54)	7.38e−05	0.001
		cg06898831	rs209474	−0.245	0.259	1.29(1.13−1.49)	2.43e−04	0.000
	BTG2	cg23371584	rs75335174	2.769	0.032	1.03(1.01−1.06)	3.77e−03	0.524
	CCL2	cg16700758	rs3091315	−0.328	0.263	1.30(1.16−1.46)	4.21e−06	0.047
		cg17864156	rs3091316	0.341	−0.256	0.77(0.70−0.86)	2.43e−06	0.045
	CHFR	cg02667335	rs2291255	−0.500	−0.129	0.88(0.81−0.95)	1.87e−03	0.093
	CSF3	cg04193923	rs4795415	−0.193	0.347	1.42(1.16−1.72)	5.23e−04	0.005
	DNMT3A	cg10614445	rs56218834	−0.313	−0.175	0.84(0.76−0.93)	7.39e−04	0.743
	EHMT2	cg02778334	rs137893789	0.377	−0.422	0.66(0.49−0.87)	3.28e−03	0.000
	ERBB2	cg05616858	rs2517953	−0.439	0.160	1.17(1.09−1.27)	3.83e−05	0.009
		cg14187895	rs2517953	−0.308	0.228	1.26(1.12−1.41)	8.95e−05	0.004
		cg26615017	rs2952155	0.263	−0.275	0.76(0.65−0.88)	4.11e−04	0.004
	ETS1	cg21695395	rs4937340	0.371	0.152	1.16(1.05−1.29)	3.26e−03	0.241
	EXOSC9	cg19748678	rs56077950	0.620	−0.077	0.93(0.88−0.97)	2.69e−03	0.462
	FBN1	cg14164380	rs55694948	−0.438	0.150	1.16(1.05−1.29)	3.88e−03	0.019
	FOXN3	cg13679772	rs28367284	−0.925	−0.095	0.91(0.85−0.97)	2.79e−03	0.563
	FOXO1	cg09792463	rs9549251	−0.431	−0.112	0.89(0.83−0.96)	2.81e−03	0.446
		cg10696351	rs12583418	−0.576	−0.084	0.92(0.87−0.97)	2.38e−03	0.455
	GPX1	cg24011261	rs7648841	−0.240	−0.666	0.51(0.41−0.64)	8.45e−09	0.993
	GPX4	cg04903600	rs62131210	0.345	−0.157	0.85(0.77−0.95)	3.04e−03	0.025
	HLA−A	cg17678719	rs2076179	0.215	0.491	1.63(1.34−1.99)	9.52e−07	0.988
	HLA−B	cg18996202	rs1362126	−0.503	−0.222	0.80(0.75−0.85)	2.04e−11	0.988
	HLA−C	cg11247343	rs114475699	−0.522	0.163	1.18(1.09−1.27)	1.45e−05	0.011
	HNF1B	cg21250756	rs12951345	0.345	0.157	1.17(1.05−1.30)	3.71e−03	0.433
	HNRNPH1	cg20740299	rs1136267	−0.503	0.104	1.11(1.04−1.18)	2.01e−03	0.142
		cg20773320	rs1136267	−0.624	0.083	1.09(1.03−1.15)	1.84e−03	0.142
	HORMAD1	cg22823121	rs11587444	0.381	−0.133	0.88(0.81−0.95)	1.58e−03	0.025
	HP	cg23815491	rs8062041	1.140	0.041	1.04(1.01−1.07)	2.90e−03	0.274
	IL6	cg13104385	rs1474347	−1.068	−0.049	0.95(0.93−0.98)	6.31e−04	0.519
		cg05265849	rs1474347	−0.953	−0.054	0.95(0.92−0.98)	6.44e−04	0.519
	IRF1	cg15375424	rs2706386	0.276	−0.434	0.65(0.55−0.76)	6.56e−08	0.909
	IRF3	cg03172226	rs12104272	0.254	0.210	1.23(1.07−1.42)	3.66e−03	0.095
	IRF5	cg12816198	rs3757387	0.571	0.144	1.15(1.09−1.22)	2.39e−07	0.000
		cg14349538	rs3757387	−0.322	−0.254	0.78(0.70−0.86)	2.44e−06	0.992
		cg26616347	rs3757387	−0.283	−0.290	0.75(0.66−0.85)	5.90e−06	0.992
		cg06630958	rs3757387	−0.221	−0.371	0.69(0.58−0.82)	3.03e−05	0.992
		cg22630713	rs3807307	−0.200	−0.409	0.66(0.54−0.81)	6.28e−05	0.992
	JAK2	cg20394284	rs10758669	0.605	0.166	1.18(1.12−1.25)	1.05e−09	0.989
	MBL2	cg19492498	rs13312736	−0.583	0.105	1.11(1.04−1.19)	2.87e−03	0.328
	MCL1	cg02961109	rs11204666	0.302	−0.251	0.78(0.66−0.91)	2.32e−03	0.022
	MED1	cg15445000	rs4286140	−0.756	0.084	1.09(1.03−1.15)	1.79e−03	0.551
		cg03013999	rs12948381	−0.503	0.122	1.13(1.04−1.22)	2.73e−03	0.550
	MICA	cg11268327	rs139063538	−1.708	0.048	1.05(1.03−1.07)	2.29e−05	0.074
		cg13139838	rs114921825	0.344	−0.239	0.79(0.69−0.90)	3.41e−04	0.985
	MIR146B	cg09701700	rs1536309	−0.509	0.098	1.10(1.04−1.18)	2.29e−03	0.050
	MUC1	cg15699386	rs2070803	−0.222	−0.243	0.78(0.68−0.91)	1.54e−03	0.782
	NOTCH1	cg13659914	rs35116034	−0.318	0.267	1.31(1.12−1.52)	5.81e−04	0.033
	NUPR1	cg01803679	rs180743	−0.271	−0.205	0.81(0.72−0.92)	7.95e−04	0.625
		cg15149645	rs4149392	−0.310	−0.161	0.85(0.77−0.94)	2.11e−03	0.615
		cg01542023	rs4149406	−0.414	−0.119	0.89(0.82−0.96)	1.60e−03	0.833
	PINK1	cg24455365	rs3121395	0.657	−0.138	0.87(0.80−0.95)	1.72e−03	0.014
	PLAU	cg04939496	rs2227551	0.453	−0.157	0.85(0.80−0.92)	2.09e−05	0.961
	POLE	cg03742729	rs4883628	0.436	0.102	1.11(1.03−1.19)	4.11e−03	0.024
		cg11808531	rs4883629	1.124	0.040	1.04(1.01−1.07)	3.46e−03	0.024
	POLR3H	cg04809988	rs17367849	0.876	0.075	1.08(1.04−1.12)	2.34e−04	0.861
		cg06481639	rs2413643	−0.246	−0.267	0.77(0.65−0.90)	1.44e−03	0.894
		cg02738086	rs12484722	−0.521	−0.125	0.88(0.82−0.95)	4.01e−04	0.338
	PSMD9	cg05989984	rs7309024	−0.204	−0.246	0.78(0.66−0.92)	3.58e−03	0.818
	PVT1	cg03566001	rs9693781	−1.013	0.075	1.08(1.03−1.12)	2.63e−04	0.868
	RNF213	cg12648893	rs8072917	−0.212	−0.255	0.78(0.66−0.92)	3.21e−03	0.015
	RTEL1	cg15788645	rs4809322	0.541	−0.177	0.84(0.75−0.94)	2.20e−03	0.017
		cg03339910	rs2297440	−0.248	0.478	1.61(1.31−1.98)	5.97e−06	0.965
		cg00622799	rs909334	−0.231	0.555	1.74(1.37−2.21)	5.34e−06	0.964
	SMAD3	cg24032190	rs17293632	0.459	0.139	1.15(1.06−1.24)	3.42e−04	0.035
	SMUG1	cg03856366	rs3136378	0.352	−0.134	0.88(0.80−0.96)	3.62e−03	0.152
	SP2	cg00458337	rs8079333	−0.974	−0.049	0.95(0.92−0.98)	2.67e−03	0.423
		cg16575821	rs2325747	−0.814	−0.057	0.94(0.91−0.98)	2.46e−03	0.423
	STAT3	cg06270615	rs6503697	−0.855	0.125	1.13(1.09−1.18)	8.74e−10	0.978
	STAT5A	cg05117208	rs28606005	−0.219	0.378	1.46(1.20−1.78)	1.89e−04	0.984
		cg20388732	rs8064638	−0.266	0.311	1.36(1.17−1.59)	7.28e−05	0.984
		cg03001305	rs16967609	0.210	−0.394	0.68(0.55−0.83)	2.40e−04	0.980
	TCEA2	cg04956806	rs57642905	−0.621	0.099	1.10(1.04−1.18)	1.65e−03	0.009
		cg27370028	rs57642905	−0.607	0.102	1.11(1.04−1.18)	1.67e−03	0.009
		cg10494860	rs58269830	−0.808	0.076	1.08(1.03−1.13)	1.60e−03	0.009
		cg24348495	rs28638160	−0.791	0.078	1.08(1.03−1.13)	1.51e−03	0.009
		cg12176783	rs7264802	−0.617	0.101	1.11(1.04−1.18)	1.31e−03	0.009
		cg02197387	rs7264802	−0.544	0.114	1.12(1.04−1.20)	1.40e−03	0.009
	TFEB	cg02284014	rs78470916	−0.870	0.120	1.13(1.04−1.22)	2.93e−03	0.011
	TRIM26	cg06400745	rs188837807	−0.368	0.333	1.40(1.23−1.59)	4.83e−07	0.051
		cg12663302	rs17184549	0.248	0.322	1.38(1.17−1.63)	1.22e−04	0.016
	TUFM	cg00348858	rs62037363	−0.285	−0.192	0.82(0.74−0.92)	9.72e−04	0.803
	UBE2L3	cg06850285	rs12169001	0.275	0.262	1.30(1.13−1.49)	2.20e−04	0.962
		cg25421647	rs2876971	0.360	0.201	1.22(1.11−1.35)	6.42e−05	0.968
		cg26979056	rs11089637	0.226	0.305	1.36(1.13−1.63)	1.12e−03	0.967
	UBQLN4	cg22796458	rs3754293	−0.453	−0.105	0.90(0.84−0.96)	2.34e−03	0.058
	UBTF	cg13780065	rs2526011	0.469	−0.181	0.83(0.75−0.93)	1.70e−03	0.021
	YWHAG	cg10167463	rs7789940	−0.777	−0.063	0.94(0.90−0.98)	2.30e−03	0.012
	ZBTB4	cg12456884	rs9675122	−0.217	0.255	1.29(1.10−1.51)	1.58e−03	0.010
		cg22685463	rs6761	0.274	−0.205	0.81(0.72−0.92)	6.72e−04	0.010

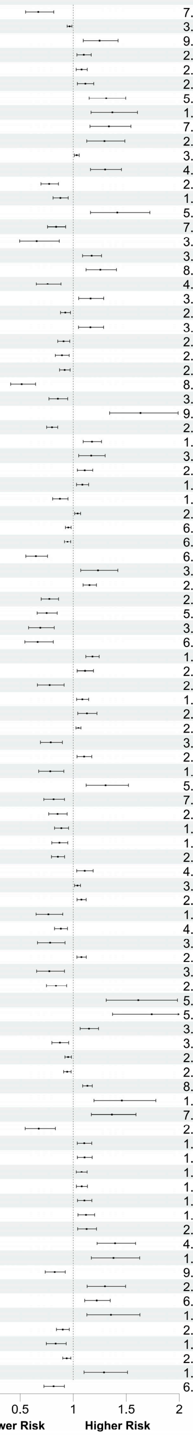

OR:odds ratio. OR＞1 means higer risk, OR＜1 means lower risk.

Figure S4. Associations of genetically predicted DDR-related genes methylation with Crohn's disease in Mendelian randomization analysis

Disease	Gene	Methylation probe	Instrumental variant	mQTL size effect	β coefficient	OR(95%CI)	P_{SMR} value	Colocalization
**Crohn's disease**								
	BAD	cg23796481	rs479552	0.536	−0.196	0.82(0.73−0.92)	8.47e−04	0.551
	BRD2	cg02448295	rs20547	1.657	0.208	1.23(1.12−1.35)	5.99e−06	0.041
	FOXK1	cg10106935	rs11973119	−0.725	−0.313	0.73(0.62−0.87)	3.19e−04	0.030
		cg06660057	rs11973119	−0.765	−0.297	0.74(0.63−0.87)	2.95e−04	0.030
		cg22753962	rs11973119	−1.022	−0.222	0.80(0.71−0.90)	2.12e−04	0.030
		cg15770585	rs11973119	−0.530	−0.429	0.65(0.51−0.83)	5.95e−04	0.030
	GPX1	cg07274523	rs34293138	−0.688	−0.162	0.85(0.78−0.93)	3.04e−04	0.908
	GTF2H4	cg02815249	rs147642505	0.226	0.801	2.23(1.51−3.30)	6.20e−05	0.007
	HLA−A	cg15319255	rs2517910	0.441	−0.243	0.78(0.68−0.90)	6.15e−04	0.988
		cg17678719	rs2076179	0.215	0.631	1.88(1.35−2.61)	1.66e−04	0.988
	HLA−B	cg18996202	rs1362126	−0.503	−0.268	0.76(0.68−0.86)	1.69e−05	0.988
	HLA−DQB1	cg09555323	rs9461776	−0.529	−0.853	0.43(0.31−0.59)	3.80e−07	0.000
		cg10846853	rs34411532	0.663	−0.298	0.74(0.66−0.84)	1.38e−06	0.039
		cg23464743	rs9274617	1.039	0.128	1.14(1.07−1.21)	9.51e−05	0.000
	NOD2	cg01068906	rs2066844	−0.988	−0.438	0.64(0.53−0.79)	2.44e−05	0.995
		cg01055281	rs2066844	−0.438	−0.988	0.37(0.22−0.64)	3.75e−04	0.995
		cg08195491	rs2066844	−1.132	−0.383	0.68(0.57−0.81)	1.95e−05	0.995
	PSMD4	cg10963543	rs61817972	−0.327	0.386	1.47(1.20−1.80)	1.88e−04	0.927
		cg08236358	rs61817972	−0.240	0.526	1.69(1.26−2.27)	4.41e−04	0.927
	STAT3	cg24718015	rs4796793	0.318	−0.415	0.66(0.52−0.83)	5.29e−04	0.459
		cg17833746	rs3736162	0.285	−0.462	0.63(0.48−0.82)	5.39e−04	0.017
		cg06270615	rs6503697	−0.855	0.151	1.16(1.07−1.26)	1.79e−04	0.490
	TRIM26	cg06400745	rs188837807	−0.368	0.449	1.57(1.25−1.97)	1.26e−04	0.063

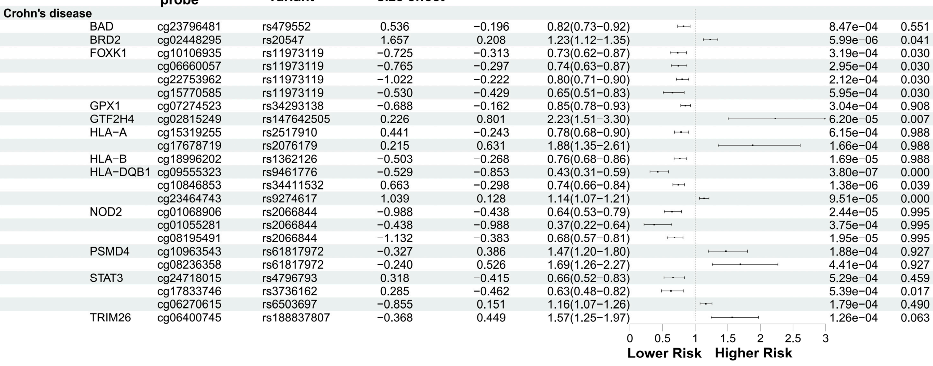

OR:odds ratio. OR＞1 means higer risk, OR＜1 means lower risk.

Figure S5. Associations of genetically predicted DDR-related genes methylation with ulcerative colitis in Mendelian randomization analysis

Disease	Gene	Methylation probe	Instrumental variant	mQTL size effect	β coefficient	OR(95%CI)	P_{SMR} value	Colocalization
ulcerative colitis								
	ACTB	cg05971148	rs852389	−0.208	−0.395	0.67(0.54−0.84)	5.20e−04	0.000
	ASCC2	cg02474858	rs737911	−0.293	0.279	1.32(1.12−1.55)	7.64e−04	0.899
	BRD2	cg26953232	rs580962	0.230	0.323	1.38(1.15−1.66)	6.93e−04	0.004
		cg17451586	rs580962	0.249	0.299	1.35(1.14−1.60)	5.43e−04	0.004
		cg06898831	rs209474	−0.245	0.284	1.33(1.12−1.57)	8.01e−04	0.003
	CCL2	cg16700758	rs3091315	−0.328	0.295	1.34(1.17−1.54)	1.88e−05	0.416
		cg17864156	rs3091316	0.341	−0.286	0.75(0.66−0.85)	1.26e−05	0.402
	CHFR	cg02667335	rs2291255	−0.500	−0.197	0.82(0.74−0.91)	1.38e−04	0.222
	CSF3	cg04193923	rs4795415	−0.193	0.395	1.49(1.17−1.88)	9.83e−04	0.016
	CYP1A2	cg01359532	rs11072508	0.374	−0.160	0.85(0.77−0.94)	2.24e−03	0.020
	ERBB2	cg05616858	rs2517953	−0.439	0.187	1.21(1.10−1.32)	8.42e−05	0.027
		cg14187895	rs2517953	−0.308	0.267	1.31(1.14−1.50)	1.72e−04	0.000
		cg26615017	rs2952155	0.263	−0.303	0.74(0.61−0.89)	1.20e−03	0.000
	ERG	cg12884169	rs8130434	1.139	−0.051	0.95(0.92−0.98)	2.88e−03	0.456
	ETS1	cg21695395	rs4937340	0.371	0.191	1.21(1.07−1.37)	2.68e−03	0.577
	FBN1	cg14164380	rs55694948	−0.438	0.194	1.21(1.07−1.38)	2.70e−03	0.020
	FOXK1	cg10888348	rs4492258	−0.710	0.157	1.17(1.08−1.27)	1.55e−04	0.068
		cg18733230	rs4492258	−0.333	0.334	1.40(1.16−1.69)	5.06e−04	0.068
	FOXN3	cg13679772	rs28367284	−0.925	−0.117	0.89(0.82−0.96)	2.71e−03	0.611
	FOXO1	cg09792463	rs9549251	−0.431	−0.144	0.87(0.79−0.95)	1.76e−03	0.580
		cg10696351	rs12583418	−0.576	−0.107	0.90(0.84−0.96)	1.61e−03	0.596
	FOXP1	cg06241812	rs4055824	−0.342	−0.189	0.83(0.73−0.93)	1.83e−03	0.708
		cg05384123	rs4055824	−0.242	−0.267	0.76(0.64−0.91)	3.17e−03	0.708
	GPX1	cg24011261	rs7648841	−0.240	−0.747	0.47(0.36−0.61)	2.18e−08	0.993
		cg05551922	rs4241406	0.193	−0.715	0.49(0.36−0.67)	5.69e−06	0.995
	GTF2H4	cg16990009	rs147438193	−2.095	−0.094	0.91(0.86−0.96)	1.43e−03	0.973
	HDAC5	cg02362984	rs2129301	−0.740	0.142	1.15(1.06−1.26)	1.31e−03	0.015
	HLA−A	cg15319255	rs2517910	0.441	−0.236	0.79(0.72−0.86)	2.46e−07	0.986
		cg17678719	rs2076179	0.215	0.455	1.58(1.27−1.95)	2.97e−05	0.986
	HLA−B	cg18996202	rs1362126	−0.503	−0.215	0.81(0.75−0.87)	5.10e−08	0.986
	HLA−DQB1	cg01745539	rs9274579	1.101	0.172	1.19(1.14−1.23)	4.14e−19	0.000
		cg22233843	rs9274573	−0.895	−0.171	0.84(0.81−0.88)	1.06e−15	0.000
		cg22984282	rs9274662	−0.965	−0.161	0.85(0.82−0.88)	2.83e−16	0.000
		cg13353717	rs9274662	−1.004	−0.155	0.86(0.82−0.89)	2.05e−16	0.000
		cg05724777	rs9274669	−0.709	−0.224	0.80(0.76−0.84)	7.10e−16	0.000
	HLA−DRB1	cg15982117	rs9271161	−1.521	−0.120	0.89(0.86−0.92)	5.22e−13	0.000
		cg10632894	rs9271413	−1.450	−0.134	0.88(0.84−0.91)	6.71e−14	0.012
		cg04601775	rs9271147	−1.051	−0.171	0.84(0.80−0.88)	4.54e−12	0.016
	IRF1	cg15375424	rs2706386	0.276	−0.444	0.64(0.54−0.77)	1.24e−06	0.925
		cg17394978	rs2706384	0.641	−0.191	0.83(0.78−0.88)	6.60e−09	0.876
	IRF5	cg12816198	rs3757387	0.571	0.169	1.18(1.11−1.27)	6.78e−07	0.001
		cg14349538	rs3757387	−0.322	−0.300	0.74(0.65−0.84)	5.08e−06	0.001
		cg26616347	rs3757387	−0.283	−0.341	0.71(0.61−0.83)	1.11e−05	0.996
		cg06630958	rs3757387	−0.221	−0.438	0.65(0.52−0.80)	4.80e−05	0.996
		cg22630713	rs3807307	−0.200	−0.473	0.62(0.49−0.79)	1.11e−04	0.996
		cg04864179	rs3757387	−0.365	−0.265	0.77(0.69−0.86)	2.85e−06	0.996
		cg00140447	rs3807306	−0.306	−0.295	0.74(0.65−0.85)	1.55e−05	0.996
	JAK2	cg20394284	rs10758669	0.605	0.163	1.18(1.10−1.25)	6.91e−07	0.995
	MAP2K1	cg00098814	rs7176821	−0.855	−0.110	0.90(0.83−0.96)	3.58e−03	0.505
		cg00319686	rs71398266	−1.922	−0.051	0.95(0.92−0.98)	3.57e−03	0.525
	MCL1	cg02961109	rs11204666	0.302	−0.307	0.74(0.60−0.90)	2.50e−03	0.034
	MIR146B	cg09701700	rs1536309	−0.509	0.124	1.13(1.05−1.22)	1.81e−03	0.023
	POLE	cg03742729	rs4883628	0.436	0.128	1.14(1.04−1.24)	3.47e−03	0.021
		cg11808531	rs4883629	1.124	0.050	1.05(1.02−1.09)	2.88e−03	0.021
	POLR3H	cg04809988	rs17367849	0.876	0.076	1.08(1.03−1.13)	2.43e−03	0.162
		cg03806693	rs1972057	−0.813	−0.080	0.92(0.88−0.97)	2.98e−03	0.540
		cg02738086	rs12484722	−0.521	−0.126	0.88(0.81−0.96)	3.13e−03	0.165
	PPP5C	cg09977703	rs62136103	1.175	−0.065	0.94(0.90−0.97)	5.32e−04	0.780
	PTCH1	cg13461049	rs28394554	−0.288	0.247	1.28(1.09−1.50)	2.82e−03	0.029
		cg03626278	rs28491365	0.459	−0.153	0.86(0.78−0.94)	1.93e−03	0.029
		cg26878949	rs28705285	−0.438	0.155	1.17(1.06−1.29)	2.30e−03	0.035
	RNF213	cg12648893	rs8072917	−0.212	−0.309	0.73(0.60−0.90)	3.50e−03	0.018
	RTEL1	cg03339910	rs2297440	−0.248	0.566	1.76(1.37−2.26)	9.94e−06	0.001
		cg00622799	rs909334	−0.231	0.683	1.98(1.47−2.66)	5.73e−06	0.961
	RXRA	cg14472716	rs12006435	0.801	−0.103	0.90(0.85−0.96)	1.94e−03	0.092
	SMAD3	cg24032190	rs17293632	0.459	0.138	1.15(1.05−1.26)	3.35e−03	0.030
	SP2	cg16575821	rs2325747	−0.814	−0.073	0.93(0.89−0.97)	1.74e−03	0.238
	STAT3	cg17833746	rs3736162	0.285	−0.346	0.71(0.60−0.84)	5.44e−05	0.981
		cg06270615	rs6503697	−0.855	0.115	1.12(1.07−1.18)	3.87e−06	0.985
	STAT5A	cg05117208	rs28606005	−0.219	0.363	1.44(1.14−1.80)	1.73e−03	0.854
		cg20388732	rs8064638	−0.266	0.299	1.35(1.13−1.61)	1.08e−03	0.854
		cg03001305	rs16967609	0.210	−0.378	0.68(0.54−0.87)	1.96e−03	0.852
		cg06357561	rs9907247	0.214	−0.371	0.69(0.55−0.87)	1.74e−03	0.849
	STAT5B	cg19465320	rs9889323	0.244	−0.314	0.73(0.60−0.89)	2.06e−03	0.953
		cg18810026	rs8079317	−0.344	0.231	1.26(1.10−1.44)	6.79e−04	0.857
	TCEA2	cg00636737	rs4408777	0.303	0.197	1.22(1.07−1.38)	2.47e−03	0.122
	TRIM26	cg06400745	rs188837807	−0.368	0.320	1.38(1.19−1.59)	1.77e−05	0.047
		cg12663302	rs17184549	0.248	0.356	1.43(1.18−1.74)	3.39e−04	0.027
	UBTF	cg13780065	rs2526011	0.469	−0.210	0.81(0.71−0.93)	2.96e−03	0.022
	YWHAG	cg10167463	rs7789940	−0.777	−0.075	0.93(0.88−0.97)	3.18e−03	0.011

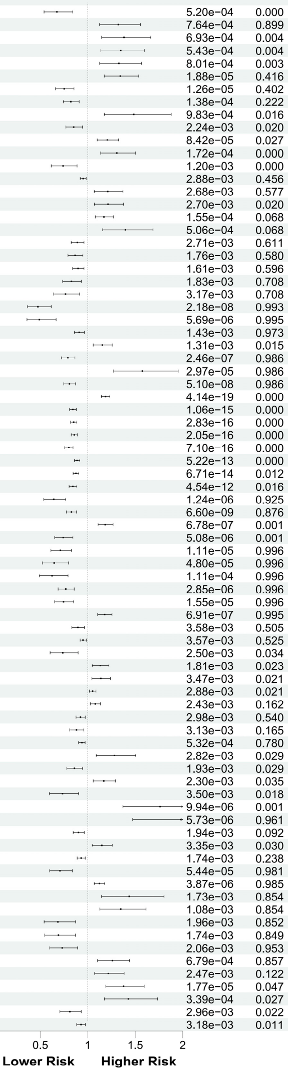

OR:odds ratio. OR＞1 means higer risk, OR＜1 means lower risk.
